# Two new species and a new combination from Zhejiang, East China

**DOI:** 10.3897/phytokeys.184.73327

**Published:** 2021-11-05

**Authors:** Yi-Fei Lu, Yue-Liang Xu, Wen-Yuan Xie, Hong-Wei Zhang, Zi-Hong Zheng, Xin Cai, Xiao-Feng Jin

**Affiliations:** 1 School of Forestry and Bio-Technology, Zhejiang Agricultural and Forestry University, Lin’an, Zhejiang 311300, China; 2 College of Life Sciences, Zhejiang University, Hangzhou, Zhejiang, 310058, China; 3 Zhejiang Museum of Natural History, Hangzhou, Zhejiang, 310014, China; 4 Monitoring Center for Forest Resources in Zhejiang, Hangzhou, Zhejiang, 310020, China; 5 Administration of Zhejiang Qingliangfeng National Nature Reserve, Lin’an, Zhejiang, 311321, China; 6 Administration of Zhejiang Jiulongshan National Nature Reserve, Suichang, Zhejiang, 323300, China; 7 Taizhou Academy of Agricultural Sciences, Linhai, 317000, Zhejiang, China; 8 School of Life and Environment Sciences, Hangzhou Normal University, Hangzhou, Zhejiang, 311121, China

**Keywords:** East China, nomenclatural novelty, seed plant, taxonomy, Zhejiang

## Abstract

As the supplement of the flora of Zhejiang, East China, two new species were described with illustrations. *Cerastiumhuadingense* Y.F.Lu, W.Y.Xie & X.F.Jin (Caryophyllaceae) differs from *C.qingliangfengicum* in having sterile stems absent, leaves sessile, petals slightly longer than sepals, and stamens slightly shorter than sepals. *Ixeridiumdimorphifolium* Y.L.Xu, Y.F.Lu & X.Cai (Asteraceae) differs from *I.beauverdianum* in having plant stoloniferous, basal leaves dimorphic, involucre 8‒10 mm long, inner phyllaries 8, and florets 7‒10. *Paraphlomissetulosa* C.Y.Wu & H.W.Li (Lamiaceae) was reviewed and morphological characters of the corolla and stamens of its type and the specimens collected in the field survey were critically examined. With barbate anthers and strongly divergent anther cells, *Paraphlomissetulosa* was transferred to *Sinopogonanthera*, and *S.setulosa* (C.Y.Wu & H.W.Li) H.W.Zhang & X.F.Jin was consequently combined.

## Introduction

Zhejiang Province, between the area of 27°06'–31°11'N, 118°01'–123°10'E, is located on the southeastern coast of China, with the whole land area taking up about 1.1% of the country. It is a territory of diverse terrain, including the most mountains in the southwestern area with the highest peak Huangmaojian (1929 m a.s.l.) in Longquan County, central hills, northern alluvial plains and numerous eastern coastal islands. It has a typical subtropical monsoon climate with marked seasonal changes and good climatic conditions. According to the latest report, there are over 4800 species of vascular plants belonging to 1587 genera in 262 families in total, showing the abundance of plant species (Jin et al. unpublished).

Since the winter of 2014, we have prepared the new edition of *Flora of Zhejiang*. Our field surveys from Zhejiang and specimen examination and identification have resulted in the discovery of 22 new species, three subspecies and four varieties of the seed plants (Chen et al. 2017; Lu et al. 2017a, 2017b, 2019; Zhang et al. 2017; Jin et al. 2018, 2019; Xu et al. 2018; Cai et al. 2019; Xie et al. 2019a, 2019b, 2019c, 2020a, 2020b). As the supplement of the flora of Zhejiang, we herein reported the novelties below.

## Results

### Cerastium huadingense

Taxon classificationPlantaeCaryophyllalesCaryophyllaceae

Y.F.Lu, W.Y.Xie & X.F.Jin sp. nov.

F833EAE4-628F-5D74-98DB-BB823A56DFEE

urn:lsid:ipni.org:names:77221580-1

**Figure 1.**
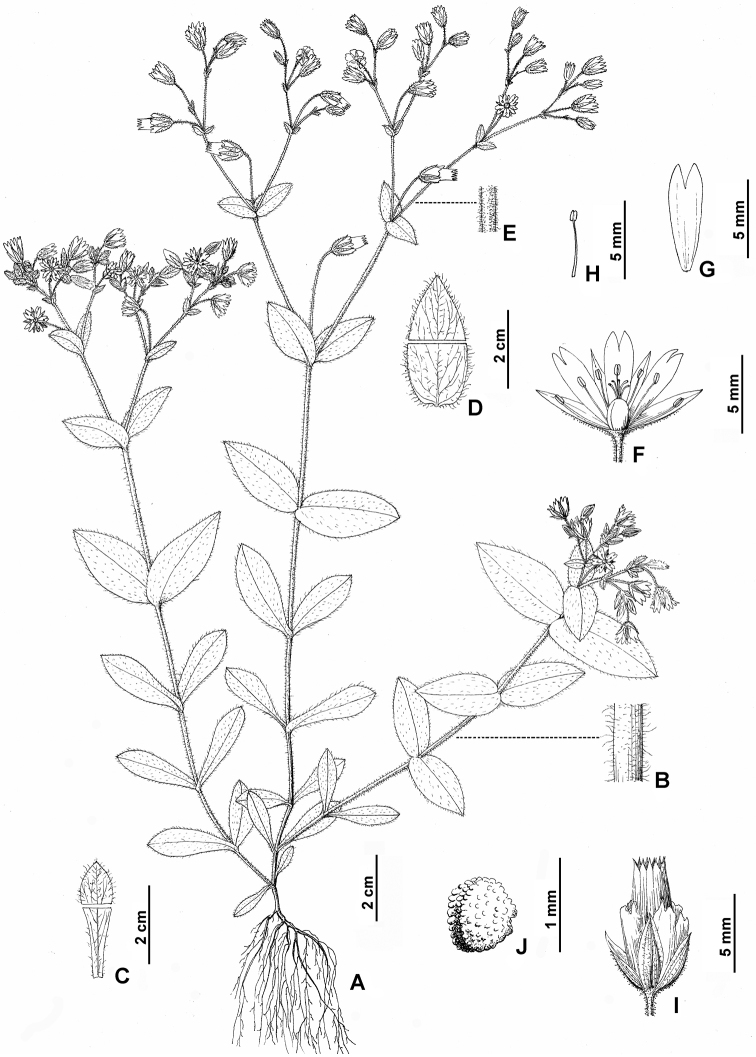
*Cerastiumhuadingense* sp. nov. **A** habit **B** indumentum on stem **C** basal leaf **D** upper leaf **E** indumentum on peduncle **F** vertical section of flower (showing sepals, petals, stamens and pistil) **G** petal **H** stamen **I** capsule with sepals and petals **J** seed (Illustrated by Hong Wang; based on *Xiao-Feng Jin & Yi-Fei Lu* 4583, ZM).

**Figure 2.**
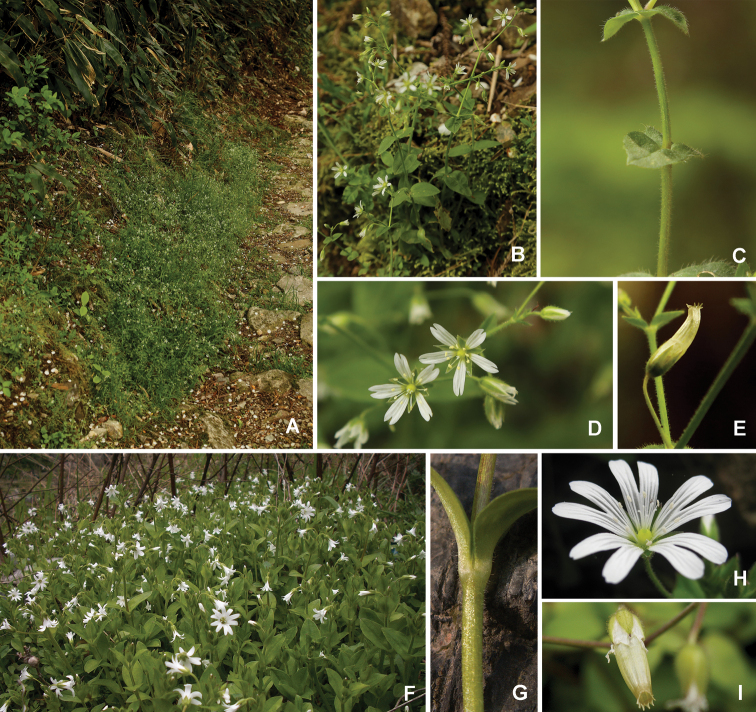
Comparison of *Cerastiumhuadingense* and *C.qingliangfengicum***A–E***Cerastiumhuadingense* sp. nov. **A** habitat **B** habit **C** indumentum on stem **D** flower **E** capsule **F–I***Cerastiumqingliangfengicum***F** habitat **G** indumentum on stem **H** flower **I** capsule.

Chinese name: 华顶卷耳 Figs 1 , 2

#### Latin diagnosis.

*Species nova haec C. qingliangfengico* H.W.Zhang & X.F.Jin *affinis est, quae caulibus sterilibus frequent praesentibus, foliis petiolatis, petiolis 5‒9 mm longis, petalis sepalis 2-plo ultra longioribus, 10‒12 mm longis, staminibus sepalis leviter longioribus differt*.

#### Type.

China. Zhejiang (浙江): Tiantai (天台), Mount Huading (华顶山), near Huading Temple (华顶讲寺), roadside at forest margin, 29°15'06.31"N, 121°05'24.52"E, alt. 1000 m, 25 April 2020, *Xiao-Feng Jin & Yi-Fei Lu* 4583 (holotype: ZM; isotypes: HTC, PE).

#### Description.

***Herb*** perennial, 12‒25 cm tall. ***Roots*** slender. ***Stems*** caespitose, unbranched, erect, green, sparsely to densely white-pubescent. ***Leaves*** opposite; basal and lower leaf blades spatulate or narrowly spatulate, 1.2‒3.2 cm long, 0.4‒0.8 cm wide, entire and sparsely ciliate at margin, apex acute, base attenuate, sparsely pubescent on both surfaces; upper leaf blades ovate, ovate-elliptic or oblong, 1.2‒4 cm long, 0.5‒1.5 cm wide, entire and sparsely ciliate at margin, apex acute, base slightly cordate, sparsely pubescent on both surfaces, sessile. ***Cyme*** terminal or axillary in upper part of stems, 6‒15-flowered; bracts leaf-like but smaller, narrowly ovate or lanceolate, herbaceous, sparsely pubescent on both surfaces; pedicels slender, 3‒12 mm long, white-pubescent; flowers white, 1.1‒1.3 cm in diam. ***Sepals*** 5, lanceolate, green, 5‒6 mm long, apex acuminate, margin membranous, abaxially pilose and glandular-pubescent. ***Petals*** 5, narrowly obovate, white, 6‒7 mm long, slightly longer than sepals, 2-lobed for ± 1/4 of length, lobes broadly ovate-oblong, apex acute, base glabrous. ***Stamens*** 10, filaments glabrous, slightly shorter than sepals. ***Styles*** 5, linear. ***Capsules*** cylindric, ± 2 as long as sepals, 10-toothed. ***Seeds*** numerous, subtriangular-globose, slightly compressed, brown, ca. 1 mm in diam., tuberculate. Flowering and fruiting April-May.

#### Distribution and habitat.

This new species is known from Mount Huading of Tiantai County and Mount Siming of Yuyao County, eastern Zhejiang. It grows under forest, in wet places or roadside at forest margin at the elevation of 900‒1000 m.

#### Phenology.

Flowering and fruiting from mid-April to mid-May.

#### Etymology.

The specific epithet ‘*huadingense*’ refers to the type locality of the new species.

#### Conservation status.

Vulnerable, VU B2ab(iii)C1 (IUCN 2019). The new species is only known from two localities, Mount Huading in Tiantai and Mount Siming in Yuyao, and occupied less than 400 km^2^ with about 5000 mature individuals. This species is considered as Vulnerable (VU) according to IUCN Red List Categories and Criteria (IUCN 2019) based on the current data.

#### Specimen examined.

Zhejiang (浙江): Tiantai (天台), Mount Huading (华顶山), Mochi (墨池), in wet place, alt. 900 m, 14 April 2018, *Wen-Yuan Xie* TT18041411 (PE, ZJFC, ZM), the same locality, under forest, 12 May 1978, *Chao-Fang Zhang* 3881 (ZJFC, ZM), roadside, alt. 1000 m, 25 April. 2020, *Xiao-Feng Jin & Yi-Fei Lu* 4584 (HTC, PE, ZM), 11 May 2020, *Yi-Fei Lu* 235 (HTC, PE, ZJFC, ZM); Mount Huading (华顶山), Ximaopeng (西茅蓬), roadside, alt. 900 m, 26 April 1976, *Chao-Zong Zheng* 8160 (HTC, ZM). Yuyao (余姚), Mount Siming (四明山), 28 April. 2018, *Zheng-Hai Chen & Jian-Sheng Wang* YY18042801 (HTC, ZJFC, ZM).

#### Notes.

The genus *Cerastium* contains ca. 100 species and is almost cosmopolitan, but is mainly distributed in north temperate regions. Twenty-five species were recorded in China (Lu and Morton 2001; Zhang et al. 2008; Yao et al. 2021). A recent study revealed that the monotypic genus *Pseudocerastium* was merged in *Cerastium* (Yao et al. 2021).

*Cerastiumhuadingense* X.F.Jin, W.Y.Xie & Y.F.Lu is most similar to *C.qingliangfengicum* in pubescent stems, 5 styles and petals longer than sepals, but differs in having sterile stems absent, leaves sessile, petals slightly longer than sepals, and stamens slightly shorter than sepals. With loosely flowered cymes, pubescent pedicels, and petals 2-lobed at the apex, the new species, *C.qingliangfengicum*, *C.jiuhuashanense* and *C.wilsonii* are similar to each another and form a complex. The morphological characters distinguished from these species are shown in Table 1.

**Table 1.** Comparisons of *Cerastiumhuadingense*, *C.qingliangfengicum*, *C.jiuhuashanense* and *C.wilsonii*.

Traits	*Cerastiumhuadingense*	*C.qingliangfengicum*	*C.jiuhuashanense*	*C.wilsonii*
**Leaf**				
Petiole	sessile on sterile stems	5‒9 mm long	basal leaves: 4‒5 mm long; upper ones: sessile	basal leaves: 3‒6 mm long; upper ones: sessile
Indumentum	sparsely pubescent on both surfaces	densely pubescent on both surfaces	sparsely pubescent on both surfaces	sparsely pubescent on both surfaces
**Flower**				
Sepal	5‒6 mm long	6‒8 mm long	5‒7 mm long	ca. 6 mm long
Petal	6‒7 mm long, slightly longer than sepals, 2-lobed for ± 1/4 of length	10–12 mm long, ca. twice as long as sepals, 2-lobed for ± 1/2 of length	10–12 mm long, ca. twice as long as sepals, 2-lobed for ± 1/2 of length	ca. 12 mm long, ca. twice as long as sepals, 2-lobed for ± 1/2 of length
Filament	glabrous	glabrous	villous on lower part	glabrous
Seed shape	subtriangular-globose	subtriangular-globose	subtriangular-globose	compressed globose

### Sinopogonanthera setulosa

Taxon classificationPlantaeLamialesLamiaceae

(C.Y.Wu & H.W. Li) H.W.Zhang & X.F.Jin comb. nov.

D01D21EE-1575-5C3B-ADBD-A843603121BF

urn:lsid:ipni.org:names:77221582-1

**Figure 3.**
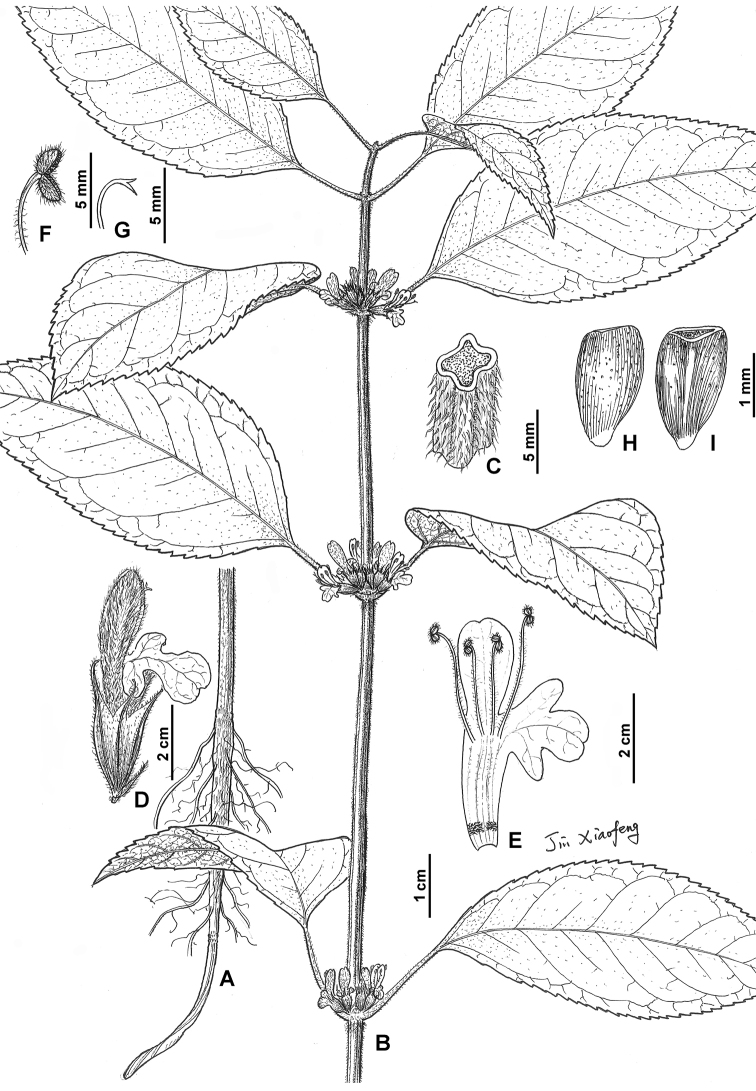
*Sinopogonantherasetulosa***A** lower part of habit **B** upper part of habit **C** indumentum on stem **D** flower with bracteole **E** opened corolla **F** anther **G** stigma **H** dorsal section of nutlet **I** ventral section of nutlet (Illustrated by Xiao-Feng Jin; based on *Yi-Fei Lu & Xiao-Feng Jin* 242, ZM).

**Figure 4.**
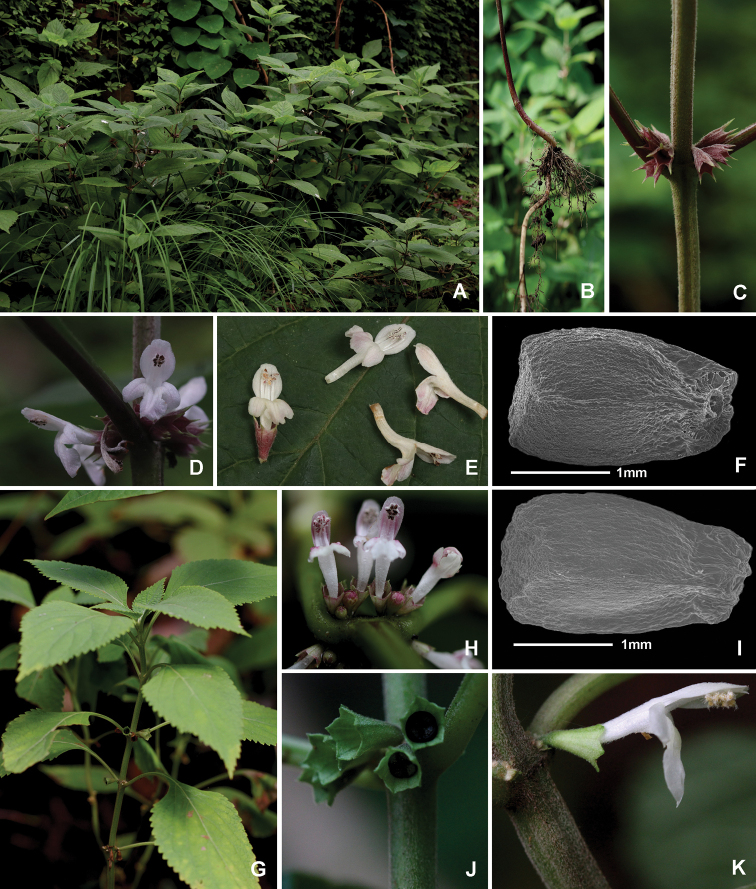
Comparison of *Sinopogonantherasetulosa* and *S.intermedia***A–F***Sinopogonantherasetulosa***A** habitat **B** rhizome **C** calyx **D** inflorescence **E** corolla **F** nutlet **G–K***Sinopogonantheraintermedia***G** habit **H** inflorescence **I** nutlet **J** calyx **K** corolla.

Chinese name: 小刺毛髯药草 Figs 3 , 4

≡ Sinopogonantherazhejiangensis H.W.Zhang & X.F.Jin, Fl. Zhejiang (New Ed.) 7: 230. 2020, nom. nud.

#### Basionym.

*Paraphlomissetulosa* C.Y.Wu & H.W.Li, Fl. Reipubl. Popularis Sin. 65(2): 602. 1977. Type: China. Anhui (安徽), Xiuning (休宁), 20 June 1959, *Ren-Hua Shan, Xiu Wu* 2226 (holotype: NAS 00072429!).

#### Note.

Based on the type specimen and the other related specimen examination, we emended the morphology of the species and described it below

#### Description emended.

***Herb*** perennial, rhizomatous. ***Stems*** erect, unbranched, 30‒80 cm tall, 2‒3.5 mm in diam., obtusely 4-angled, shallowly canaliculate, densely retrorsely white-setulose. ***Leaves*** opposite; blades elliptic, oblong-ovate, elliptic-ovate or ovate, 7‒19 cm long, 3‒9.5 cm wide, apex acuminate, base broadly cuneate, margin serrate, adaxially green, abaxially pale green, sparsely pubescent on both sides, densely pubescent on mid-ribs, lateral veins 5‒8 pairs; petioles 0.5‒4.5 cm long, adaxially flat, densely white-setulose. ***Verticillasters*** axillary, (3‒)7‒14-flowered, 1.5‒3 cm in diam.; pedicels 1‒2 mm long or subsessile; bracteoles subulate, 1.5‒3 mm long, densely setuloae. ***Calyx*** tubular-obconical, 7‒9 mm long, outside densely setulose, 5-veined, 5-lobed; teeth oblong-lanceolate or triangular, 2.5‒3 mm long, apex acuminate, white-setulose. ***Corolla*** pale purple or pink, 10‒15 mm long, outside densely appressed-pilose, inside sparsely pilose, hairy-annulate on lower part; tube 6‒8 mm long, nearly straight, upper part slightly curved, amplified, with limb 2-lipped; upper lip straight, obovate-oblong, 5‒5.5 mm long, apex emarginate; lower lip 3-lobed, middle lobe broadly obovate, ca. 3.5 mm long, 2.5‒3 mm wide, apex emarginate, lateral lobes ovate, ca. 2.5 mm long, ca. 1.5 mm wide. ***Stamens*** 4, didynamous, nearly included, posterior 2 inserted in lower lip of corolla larynx, anterior 2 inserted in upper lip of corolla larynx, filaments sparsely pilose, anthers barbate, cells strongly divergent. ***Ovary*** glabrous; style slender, slightly exserted, apex 2-cleft. ***Disc*** persistent, cup-shaped. ***Nutlets*** obovoid, obtusely trigonous, gray-brown, ca. 2 mm long, apex truncate. Flowering and fruiting June-August.

#### Specimen examined.

Anhui (安徽), Xiuning (休宁), 29 June 1959, *Ren-Hua Shan*, *Xiu Wu* 2674 (paratype: NAS). Zhejiang (浙江): Kaihua (开化), Mount Gutian (古田山), roadside under forest, alt. 400 m, 28 June 2018, *Xiao-Feng Jin* 4230 (HTC, ZM). Lin’an (临安), Tuankou Town (湍口), Tangli Village (塘里村), in grasses under *Torreya* forest, alt. 550 m, 4 August 2014, *Xiao-Feng Jin* 3316, 3317, 3318 (ZJFC, ZM), the same locality, under forest, 5 June 2013, *Hong-Wei Zhang* 0001 (HTC, ZM), cultivated in Changhua (昌化), introduced from Tangli Village (塘里村) of Tuankou Town (湍口镇), 11 June 2021, *Yi-Fei Lu & Xiao-Feng Jin* 242 (holotype: ZM; isotypes: HTC, KUN for *Sinopogonantherazhejiangensis*). Qujiang (衢江), Qianligang (千里岗), 22 July 2017, *Zheng-Hai Chen* s.n. (HTC).

#### Notes.

Twenty-eight species with nine varieties within the genus *Paraphlomis* are currently recognized, which are mainly distributed in tropical and subtropical evergreen and mixed forests (Chen et al. 2021). Li (1965) revised *Paraphlomis* from China and 21 species with eight varieties were recognized. Wu & Li (1977) published *Paraphlomissetulosa* and *P.reflexa* as two new species, and they described that *Paraphlomissetulosa* had anther cells strongly divergent. The taxonomic treatment of the genus *Paraphlomis* in *Flora of China* was just the same as Wu & Li in *Flora Reipublicae Popularis Sinicae* (Li & Hedge 1994). Guo (1993) established a new genus *Pogonanthera* close to *Paraphlomis* but mainly differs from barbate anthers and filament appendages at the apex. Unfortunately, *Pogonanthera* H.W.Li & X.H.Guo is a later homonym and Li (1993) proposed *Sinopogonanthera* H.W.Li as the replacement name, which were accepted and adopted (Guo 1995; Zheng 2005).

The genus *Sinopogonanthera* contains three species, and the key to these three species is shown as follows. *Sinopogonantheracaulopteris* H.W.Li has verticillasters up to 30-flowered, leaf blade long-elliptic, base cuneate and decurrent, petioles very short or subsessile, and stems narrowly alate. *Sinopogonantheraintermedia* (C.Y.Wu & H.W.Li) H.W.Li has verticillasters 10‒14-flowered, leaf blade ovate to ovate-rounded, base broadly cuneate, petioles 1‒6 cm long. *Sinopogonantherasetulosa* is somewhat similar to *S.intermedia* in attenuate but not decurrent leaf blades at the base and leaf blade petiolate, but differs in having calyx lobes oblong-lanceolate, 2.5‒3 mm long, corolla outside densely appressed-white-pilose, and leaves without glands. The species *Sinopogonantherazhejiangensis* (nom. nud.) is consistent to *S.setulosa* and here reduced to synonym.

### Key to the species of *Sinopogonanthera*

**Table:** 

1	Stems narrowly alate; leaf base cuneate and decurrent, petioles very short or subsessile; verticillasters 12‒30-flowered	***S.caulopteris***
–	Stems obtusely tetragonal; leaf base broadly cuneate or attanuate, but not decurrent, petioles 0.5‒6 cm long; verticillasters 3‒14-flowered	**2**
2	Calyx lobes broadly triangular, ca. 1 mm long, outside densely pubescent; corolla outside sparsely puberulent and glabular	***S.intermedia***
–	Calyx lobes oblong-lanceolate, 2.5‒3 mm long, outside densely setulose; corolla outside densely pilose	***S.setulosa***

### Ixeridium dimorphifolium

Taxon classificationPlantaeAsteralesAsteraceae

Y.L.Xu, Y.F.Lu & X.Cai sp. nov.

F68073AC-5F99-5B61-85F7-FBC08360EA49

urn:lsid:ipni.org:names:77221583-1

**Figure 5.**
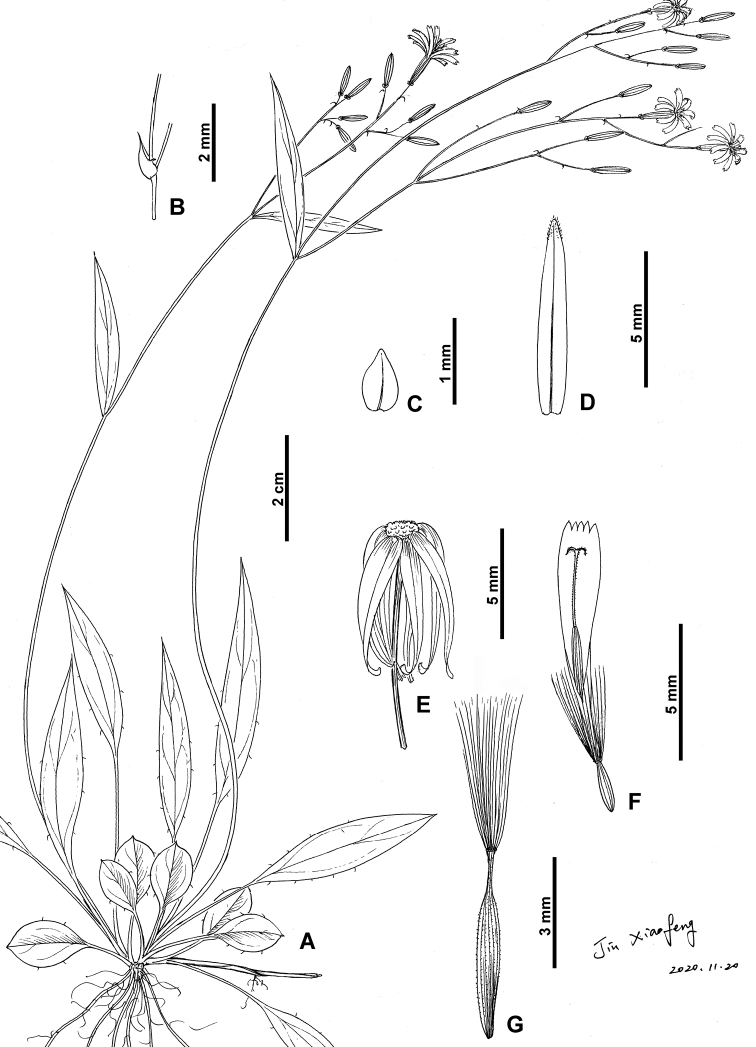
*Ixeridiumdimorphifolium* sp. nov. **A** habit **B** bract **C** outer phyllary **D** inner phyllary **E** opened involucre (showing receptacle) **F** floret **G** achene (Illustrated by Xiao-Feng Jin; based on *Xiao-Feng Jin* 4568, ZM).

**Figure 6.**
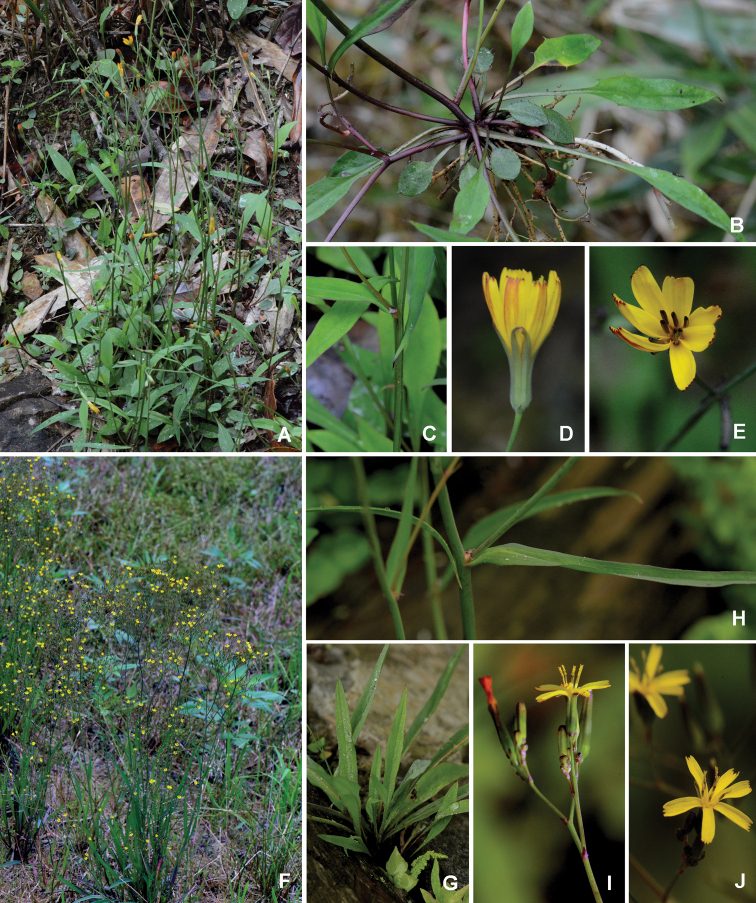
Comparison of *Ixeridiumdimorphifolium* and *I.beauverdianum***A–E***Ixeridiumdimorphifolium* sp. nov. **A** habit **B** basal part (showing dimorphic leaves and stolon) **C** cauline leaves **D** capitula (showing involucre) **E** capitula (showing florets) **F–J***Ixeridiumbeauverdianum***F** habit **G** basal part (showing monomorphic leaves) **H** cauline leaves **I** capitula (showing involucre) **J** capitula (showing florets).

Chinese name: 二型叶小苦荬 Figs 5 , 6

#### Latin diagnosis.

*Species nova I. beauverdiano* (H. Lév.) Spring. *affinis est, sed planta stolonibus, foliis basilaribus dimorphis, involucris 8‒10 mm longis, phyllis intimis 8, flosculis 7‒10 differt*.

#### Type.

**China.** Zhejiang (浙江): Suichang (遂昌), Jiulongshan National Nature Reserve (九龙山国家级自然保护区), Yangmei Keng (杨梅坑), in grasses along streams, alt. 550 m, 17 April 2020, *Xiao-Feng Jin* 4568 (holotype: ZM; isotypes: HTC, ZM).

#### Description.

***Herb*** perennial, glabrous totally. ***Stolons*** elongate, creeping. ***Stems*** erect, 20‒35 cm tall, slender, with sparse branches from base. ***Basal leaves*** crowed, present at anthesis, dimorphic; blades of early ones nearly orbicular to long-elliptic, 0.9‒2.5 cm long, 0.7‒1.3 cm wide, apex obtuse and mucronate, base broadly cuneate to rounded, margin entire and with sparsely slender ciliate-teeth, with petioles 1‒3 cm long; blades of later ones narrowly elliptic to lanceolate, 3‒6 cm long, 0.6‒1.1 cm wide, apex acute, base attenuate to a 2‒3 cm long petiole, margin entire or with sparsely slender ciliate-teeth below. ***Cauline leaves*** 1‒3; blades linear-lanceolate, 2.5‒7.5 cm long, 0.4‒0.7 cm wide, apex acuminate, base attenuate, margin entire and with/without sparsely slender ciliate-teeth below. ***Synflorescence*** corymbiform, with 6‒16 capitulae; capitula with 7‒10 florets, base with slender, long peduncle. ***Bracts*** small, broadly ovate-triangular, ca. 1 mm long, margin entire or with 1-pair ciliate-teeth. ***Involucre*** narrowly cylindrical, 8‒10 mm long. ***Phyllaries*** 2 series, abaxially glabrous; outer phyllaries broadly ovate, 0.5‒0.7 mm long, apex obtuse; inner phyllaries 8, linear-lanceolate, 7‒9 mm long, apex obtuse, short-ciliate. ***Receptacle*** flattened, glabrous, alveolate. ***Florets*** 7‒10, yellow, corolla 10‒11 mm long, tube 3‒3.5 mm long, ligule linear, apex 5-lobed. ***Achenes*** brown or pale brown, narrowly fusiform or linear-fusiform, slightly compressed, 4.5‒5 mm, finely spiculate, with ribs finely spiculate, apex attenuate to a 1‒1.5 mm long slender beak. ***Pappi*** pale brown, ca. 5 mm long, scabrid. Flowering and fruiting April‒ July.

#### Distribution and habitat.

*Ixeridiumdimorphifolium* is currently known from only two localities in Suichang and Qingyuan counties, southwestern Zhejiang Province, East China. It grows in grassy roadside along stream or wetland at the elevation of 500‒1450 m.

#### Phenology.

Flowering and fruiting from mid-April to mid-July.

#### Etymology.

The specific epithet ‘*dimorphifolium*’ refers to two different shapes of basal leaves.

#### Conservation status.

Endangered, EN B2ab(iii) (IUCN 2019). The new species is only known from two localities, and occupied less than 100 km^2^ with about 1000 mature individuals. This species is considered as Endangered (EN) according to IUCN Red List Categories and Criteria (IUCN 2019) based on the current data.

#### Specimen examined.

Same locality as type, alt. 500 m, 5 May 2017, *Yue-Liang Xu* 234, 239, 274 (ZM), alt. 550 m, 17 April 2020, *Xiao-Feng Jin* 4569 (HTC, ZM). Qingyuan (庆元), Huangpi (黄陂), in wetland, alt. 1450 m, 12 July 2020, *Xiao-Feng Jin*, *Yi-Fei Lu & Dan-Qi Liu* 4600 (HTC, ZM).

#### Notes.

*Ixeridium* (A. Gray) Tzvel. is a moderatedly sized genus within the tribe Cichorieae. It contains 15 species and distributes in E and SE Asia, with eight species occurring to China (Shi & Kilian, 2011). The genus *Ixeridium* is most morphologically similar to *Ixeris* (Cass.) Cass., but is distinguished in having achenes with 9‒12 prominent but not wing-like ribs (vs. 10 prominent and wing-like ribs) (Shih, 1997). *Ixeridiumdimorphifolium* is similar to *I.beauverdianum* in having the cauline leaf blades linear to linear-elliptic, margin nearly entire, and pappi brown or pale brown, but differs from the latter in its dimorphic basal leaves, involucres longer, 8‒10 mm long, inner phyllaries 8, florets 7‒10, and plant with stolons.

**Figure 7.**
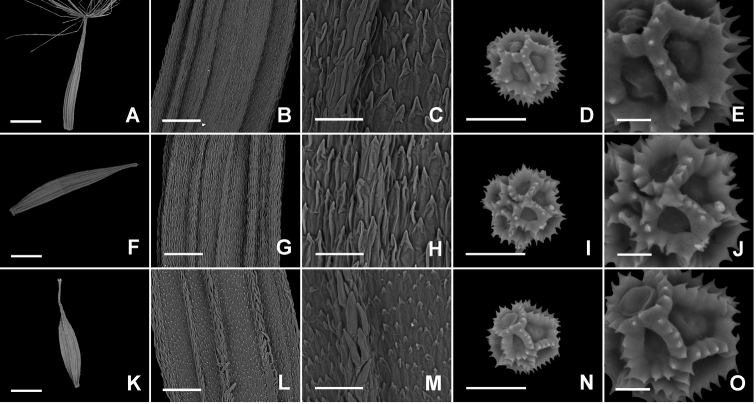
Micromorphology of achenes and pollen grains of *Ixeridiumdimorphifolium* and *I.beauverdianum***A–E***Ixeridiumdimorphifolium* sp. nov. (from Suichang) **F–J***Ixeridiumdimorphifolium* sp. nov. (from Qingyuan) **A** and **F** achene shape (scale bar: 1mm) **B** and **G** middle part (scale bar: 200μm) **C** and **H** spicules on surfaces and ribs (scale bar: 50μm) **D** and **I** pollen grains (scale bar: 20μm) **E** and **J** surface sculpture (scale bar: 5μm) **K–O***Ixeridiumbeauverdianum***K** achene shape (scale bar: 1mm) **L** middle part (scale bar: 200μm) **M** spicules on surfaces and ribs (scale bar: 50μm) **N** pollen grains (scale bar: 20μm) **O** surface sculpture (scale bar: 5μm).

Morphology of pollens and achenes of the species in Asteraceae has taxonomic significance and sometimes was used to identify similar species (Biyiklioğlu et al. 2018; Joujeh et al. 2020; Nurgül et al. 2021). The micromorphology of achenes and pollen grains of *Ixeridiumdimorphifolium* and *I.beauverdianum* was shown in Figure 7 and Table 2. The new species, *Ixeridiumdimorphifolium*, has the achenes narrowly fusiform or linear fusiform, with surfaces and ribs both finely similar spiculate, apex gradually narrow to a beak. While those of *I.beauverdianum* are fusiform, with spicules on surfaces shorter than those on ribs, apex abruptly narrow to a beak. The pollen grains of *Ixeridiumdimorphifolium* and *I.beauverdianum* are similar to each other.

**Table 2.** Comparisons of the achenes and pollen grains among *Ixeridiumdimorphifolium* (two samples) and *I.beauverdianum*.

Traits/species	*Ixeridiumdimorphifolium* (*Suichang*)	*I.dimorphifolium* (*Qingyuan*)	*I.beauverdianum*
**Achene**			
shape	narrowly fusiform, slightly compressed	linear-fusiform, slightly compressed	fusiform, slightly compressed
length	4.98±0.42 mm	5.54±0.60 mm	4.72±0.34 mm
surface	finely spiculate, with ribs finely spiculate	finely spiculate, with ribs finely spiculate	finely spiculate, with ribs finely spiculate
rib	with spicule similar to surface	with spicule similar to surface	with spicule longer than those on surface
apex	gradually narrow to a beak	gradually narrow to a beak	abruptly narrow to a beak
**Pollen**			
shape	tricolporate, spherical	tricolporate, spherical	tricolporate, spherical
size	27.55±3.60 μm	26.70±2.84 μm	24.62±1.95 μm
surface	echinophate	echinophate	echinophate

## Supplementary Material

XML Treatment for Cerastium huadingense

XML Treatment for Sinopogonanthera setulosa

XML Treatment for Ixeridium dimorphifolium
